# The impacts of introducing online postal self-sampling for sexually transmitted infections on the sustainability and equity of sexual health systems: lessons learned from a multi-method UK-wide realist evaluation

**DOI:** 10.1186/s12916-026-04908-7

**Published:** 2026-05-18

**Authors:** Jessica Sheringham, Geoff Wong, Tommer Spence, Oliver Stirrup, Anna Tostevin, Alison Howarth, Andrew Copas, David Crundwell, Louise Jackson, Catherine H. Mercer, Hamish Mohammed, Vanessa Apea, Sara Day, Jonathan D. C. Ross, Ann Sullivan, Vicky Tittle, Andrew Winter, Claire Dewsnap, Jo Gibbs, Fiona Burns

**Affiliations:** 1 Institute of Epidemiology and Health Care, University College London, London, England; 2 Nuffield Department of Primary Care Health Sciences, University of Oxford, Oxford, England; 3 Health Services Management Centre, University of Birmingham, Birmingham, England; 4 Institute for Global Health, University College London, London, England; 5 Lay representative, London, England; 6 Department of Applied Health Sciences, University of Birmingham, Birmingham, England; 7 Blood Safety, Hepatitis, STIs and HIV Division, UK Health Security Agency, London, England; 8 Barts Health NHS Trust, London, England; 9 Chelsea and Westminster Hospital NHS Foundation Trust, London, England; 10 University Hospitals Birmingham NHS Foundation Trust, Birmingham, England; 11 NHS Greater Glasgow and Clyde, Glasgow, Scotland United Kingdom; 12 Sheffield Teaching Hospitals NHS Foundation Trust, Sheffield, England

**Keywords:** Sexually transmitted infections, Realist evaluation, Digital, System transformation, Equity

## Abstract

**Background:**

Online postal self-sampling (OPSS) for sexually transmitted infections and blood borne viruses (STI/BBVs) has been introduced in several countries, because it may lower costs, and increase access for service users. There are gaps in the evidence on the equitable delivery to underserved populations beyond men who have sex with men, the implementation and maintenance of programmes and its impact on wider health systems. This study synthesised evidence from the ASSIST research programme, which evaluated the implementation, equity, impacts and economic consequences of OPSS in England (2015–2022), with a focus on understanding how and why unintended consequences may emerge in other contexts.

**Methods:**

A synthesis using a realist logic of analysis was undertaken across multiple sources of evidence from ASSIST workstreams. Context-mechanism-outcome configurations were developed from a systems perspective. They were iterated through comparisons with initial programme theory and through feedback obtained from sexual health providers, funders, researchers and service users.

**Results:**

In England, OPSS appears convenient and achieved higher testing uptake for lower costs per diagnosis. However, there were unintended consequences, principally: (1) difficulties in containing demand for OPSS could result in higher total costs than planned; (2) the introduction of OPSS affected wider sexual health system sustainability, particularly when the context changed; (3) inequalities in sexual health may widen after the introduction of OPSS in part due to missed presentation opportunities. These challenges prompted adaptive responses in health systems, leading to a rebalancing between OPSS and clinic-based services.

**Conclusions:**

While the introduction of OPSS in the context of reducing sexual health budgets offers some clear advantages to entirely clinic-based sexual health services, excessive demand and difficulties in access and use risk destabilising sexual health systems and worsening inequalities. Strategic rebalancing of OPSS and clinic-based provision is essential to mitigate unintended effects and ensure equitable, resilient service delivery.

**Protocol:**

https://doi.org/10.1136/bmjopen-2022-067170

## Background

The global burden of sexually transmitted infections and blood borne viruses remains high, with persistent inequalities [1, 2]. In parallel, many countries such as the UK are also facing reducing budgets for sexual health prevention, diagnosis and treatment. In response, numerous countries are introducing online postal self-sampling (OPSS), a digital health intervention for sexual health where service users order a self-sampling kit online, send their samples to a laboratory and receive their results remotely [3]. The World Health Organisation recommends that self-sampling is provided alongside clinic-based sexual health services to improve uptake of chlamydia and gonorrhoea testing [2]. Globally, OPSS is at varying stages of development, with heterogeneous implementation models. It has been operational in Europe, including the UK [4], and in parts of the USA [5], and Canada for several years [6].

Evidence suggests that OPSS can be acceptable to service users and may increase uptake of testing [3, 7], and may cost less to deliver than clinic-based testing [7, 8]. However, there is little evidence about the impact of introducing OPSS beyond uptake or its effects on the wider sexual health system.

ASSIST (Assessing the impact of online postal self-sampling for sexually transmitted infections on health inequalities, access to care and clinical outcomes in the UK) was a national, realist evaluation of OPSS in the UK [9]. It comprised an impact evaluation focused on health inequalities, access to care and public health outcomes, an economic evaluation, and an implementation evaluation. Our data from these evaluations within ASSIST confirmed that OPSS usage was high, the costs for asymptomatic testing lower than clinic-based testing and was generally popular with service users (Table 1). Our data also pointed to some unintended consequences that may arise with OPSS.

This paper synthesises multiple sources of quantitative and qualitative data from the ASSIST evaluation of OPSS to:

- describe and explain why, how, for whom and in what contexts unintended consequences can arise from the introduction of OPSS services within a complex system;
- compare the influences of different interactions within and between complex systems across the three case study areas (CSAs) in the UK, and their effects on the sustainability of sexual health service delivery;
- provide recommendations for planning and delivering OPSS within an equitable and sustainable sexual health system.

**Table 1:** Evidence on which this paper is based

Paper	Data Source	Method of analysis	Summary of findings
WORKSTREAM 1: Impacts on health inequalities, access to care and public health outcomes			
1.1. Impact of online postal self-sampling on changes in testing activity and other key clinical and public health outcomes [18]	Pseudonymised data from online sexual health providers in three CSAs, 2014–2022 (population covered: 3.4m)	Quantitative examination of chlamydia, gonorrhoea and HIV testing activity, new diagnoses of STI/BBVs, treatment receipt. All outcomes compared over time, by CSA and by demographic characteristics	- Introduction of OPSS into the sexual health economy led to an increase in overall testing activity - Greatest relative increase in testing activity occurred in populations that already had greater testing activity prior to OPSS (e.g. men who have sex with men (MSM)). - OPSS accounted for most new chlamydia diagnoses in 2022 in two of the CSAs. - It is difficult to track a user journey across hybrid online and clinic-based settings
1.2. How OPSS shaped access to STI services [19]	100 service user interviews, in three CSAs, 2021–2023	Qualitative thematic analysis using Levesque et al’s conceptual framework of access to healthcare [13]	- High levels of satisfaction with all but blood sampling - Participants usually able to engage with OPSS, finding it easy to use and reliable, although blood self-sampling was challenging for most. - Participants valued the support offered by clinic-based services beyond STI testing, including the opportunity to access contraception or ask staff questions, and felt this was more appropriate when they had concerns about their sexual health, such as symptoms
1.3. Equity of online and clinic testing over time [19]	National chlamydia testing in surveillance data (GUMCAD, CTAD), England 2015–2022 (n = c25m tests)	Descriptive quantitative analysis and bivariate and multivariable logistic regression to examine associations with testing mode, site and sociodemographic characteristics	- Between 2015 and 2022, OPSS testing increased from 2·6% to 38·4% of all clamydia testing; - OPSS test-positivity decreased in men from 9·3% to 7·5%) and increased in women from 8·3% to 10·6%; - People in most deprived areas least likely to use OPSS.
1.4. Online postal self-sampling testing cascade [28, 29]	Pseudonymised routine data from online sexual health providers in three CSAs, 2022	Analysis of the timeliness and completeness of each stage of the STI testing cascade, i.e. - kit orders, - all kits returned - returns with processable urine and blood samples - Outcomes Analysis undertaken by site and sociodemographic characteristics	- Variations in the timeliness and return of tests by CSA - Socio-demographic variations in return of processable samples from OPSS, e.g. individuals living in least deprived areas more likely than those in most deprived areas to return processable samples. - Low positive predictive value (4.4–7.1%) for HIV testing after excluding people known to be living with HIV across the 3 CSAs.
WORKSTREAM 2: Economic analysis			
2.1 Economic evaluation of online postal self-sampling (OPSS) and clinic-based screening for sexually transmitted infections (STIs): a case study analysis (Jackson et al. 2025 unpublished)	Pseudonymised data from online sexual health providers in three CSAs, 2014–2022 (population covered: 3.4 m) and cost data.	Economic evaluation of the costs and benefits associated with online and in clinic testing from the perspective of the sexual health provider.	OPSS services were associated with lower total costs than clinic-based services under a range of scenarios. The cost per diagnosis and infection treated was also lower with OPSS for chlamydia and gonorrhoea than clinic-based pathways but the differential in costs is reduced compared with cost per test. The costs – per test or per diagnosis –need to be interpreted with caution because they don’t capture the costs of setting up the pathways or the lost opportunities for delivering sexual health prevention services. When taking into account the treatment outcomes, the cost differences between OPSS and clinic-based testing became smaller particularly for HIV and syphilis. When considering positivity rates and treatment outcomes, OPSS is likely to be less effective but with a lower cost.
WORKSTREAM 3: Evaluation of implementation process			
3.1 Implementation of OPSS [11]	Documentary sources (n = 86, 2012–2023); staff and stakeholder interviews (n = 60), and observations (n = 12) in three CSAs, 2022–2023	Qualitative analysis using Normalisation Process Theory as principal guiding framework	- OPSS was part of major system changes. - Implementation was an ongoing process in response to changes in context. - Financial pressures and organisational relationships determined the implementation strategies available to decision-makers, how these strategies were enacted and, in turn, led to different implementation outcomes at different time points. - The COVID-19 pandemic had profound but divergent effects on OPSS implementation in each area
3.2 What lessons can be learned from how digital sexual health has been implemented in the UK – and how can we use this information to do it better / to optimise access to care, user experience, and service delivery? British Association for Sexual Health and HIV Conference June 2022, Sheffield. (Gibbs et al. [9] unpublished)	Joint symposium with presentations from ASSIST and Sequence Digital [28], implementers in four UK jurisdictions and comments from conference attendees clinicians, commissioners and academics)	Notes taken by 4 observers on presentations and audience questions/ comments, discussed and summarised after symposium by ASSIST and Sequence Digital co-investigators	Context and implementation models across devolved nations very different (e.g. Wales rapidly set up during COVID-19, in Northern Ireland noted OPSS was introduced amidst a culture of significant stigma around sex: nearly 50% of OPSS users had never used a clinic). Lots of unknowns due to little evaluation so far, including who OPSS works best for. Discussion focused on: - Whether using OPSS would result in fewer opportunities for partner notification, education, risk reduction - Workforce planning and changes needed to training to equip future doctors to work in a digital environment. - Integration of data across clinics and OPSS platforms

## Methods

### Design

We synthesised data across all ASSIST’s workstreams using a realist logic of analysis. The aim was to develop programme theory to explain for whom and in what circumstances OPSS led to unintended consequences [10]. We anticipated that different OPSS services, in different settings and delivered in diverse ways, would likely produce different outcomes (intended and unintended). OPSS models and implementation changed significantly during the study, in part influenced by major events (COVID-19 and Mpox) and in part by changing perspectives of all commissioners, providers and service users. Realist evaluation is an ideal approach to use for such heterogenous intervention implementation in rapidly changing contexts [10].

We summarise the ASSIST workstream methods and setting briefly here, with further detail available in the ASSIST protocol [9].

### Setting and programme

ASSIST evaluated OPSS, in the context of UK sexual health systems, with a focus on three case study areas (CSAs). The CSAs, summarised in Table 2 and reported in more detail in ASSIST publications [11, 12], were diverse in their size, implementation models and population characteristics, and all provided testing for sexually transmitted infections and blood borne diseases including chlamydia, gonorrhoea, HIV, and syphilis.

**Table 2:** Summary of case study area characteristics

Characteristic	CSA1	CSA2	CSA3
Population size covered	C1.1m	C8m	C450,000
Date OPSS introduced	08/2015	02/2018	01/2020
OPSS model	In-house laboratory testing and results feedback	Bespoke external testing and results system	External ‘off the shelf’ testing and results system
Provider model [11]	Integrated provider of both clinic and OPSS	External provider commissioned by consortium of local authorities	External provider subcontracted through clinical provider
Uptake in the 1st year of delivery [12]	Gradual	Increased month by month	Increased sharply from month 3, March 2020, coinciding with lockdown due to COVID19

### Theories and perspectives informing this paper

The following programme and substantive theories, and perspectives informed development of the realist programme theory of the unintended consequences of OPSS. In terms of our realist perspectives, we considered **context** to represent features of the situations into which OPSS was introduced that affect the operation of OPSS mechanisms [10]. We considered **mechanisms** as underlying processes or structures that operate in certain contexts that can explain why and how outcome patterns occur. In ASSIST’s evaluation, often these relate to agents’ (service commissioners, providers, service users) responses to a change [13] We considered **outcomes** as observable changes, which in ASSIST, were the stimulus to enquiring how and why such outcomes were observed.

#### ASSIST logic model

A logic model of how OPSS might achieve its benefits was developed by the study co-investigators (Additional File 1, slide 1). The overall design of ASSIST and each workstream was informed by this logic model. Analyses of qualitative data within the workstreams and staff and stakeholder data were also informed by specific theories relevant to their research questions, i.e. equity for service user data [14] and normalisation process theory [15] for OPSS implementation.

#### Initial programme theories to surface assumptions about OPSS and how it might achieve its outcomes

Implicit in the logic model were some assumptions underpinning the rationale for OPSS. Making these assumptions explicit was needed to underpin the realist evaluation. Therefore, programme theories from a service user and provider/commissioner perspective were developed to set out initial assumptions about the potential benefits of OPSS (Additional File 1, Slide 2).

#### Integration of a complex systems lens approach

Initially we considered OPSS as a digital innovation and developed context-sensitive causal statements that take the form of context-mechanism-outcome (CMO) configurations from this perspective (Additional File 1, Slides 3-6). However, over the course of the study we realised we were not sufficiently framing OPSS within the wider sexual health system transformations and the events and policies that shaped them. This perspective was reinforced by feedback given in response to sharing our emerging theories with stakeholders working within sexual health systems. We therefore incorporated a systems lens to our CMO configurations. This led to greater recognition of the interaction between OPSS and sexual health systems and the potential for influential interactions both within and between sexual health systems. In taking a systems perspective in ASSIST, we considered the ‘system’ as publicly funded sexual health commissioning and delivery within a regional footprint, with permeable boundaries recognising these systems were affected by wider healthcare system changes (e.g. national reconfiguration of health and public health), societal digital transformation, and major events such as COVID [9, 11]. We recognised the importance of system histories to ASSIST, i.e. changes in the configuration of the national health care and public health system in England influenced the context of ASSIST, e.g. the shift of commissioning sexual health services to local authorities in England, reductions in funding amidst increasing service demand that occurred before OPSS was introduced [9, 11].

Informed by Moore et al. [16], it also led us to consider whether OPSS could trigger other changes which may amplify the initial effect or lead to its discontinuance [16]. We included within our CMOs reactions in the system to reinforce or rebalance OPSS to align with the systems perspective of feedback loops and reactions to changes that occurred when OPSS was introduced into sexual health systems. In these linked CMOs we were guided by Jagosh et al. [17] in depicting outcomes in initial CMOs as contexts for CMOs that emerged in reaction to earlier outcomes [17].

### Data and materials

This paper drew on evidence from each ASSIST workstream, described in papers summarised in Table 2, the authors’ experiences of working in this field, and from dissemination events with stakeholders where discussion was encouraged.

### Analysis and synthesis

Analysis of raw data was undertaken separately in each workstream, summarised in Table 2 and the ASSIST protocol [9]. Interim analyses across workstreams were presented in co-investigator meetings. These interim findings were compared against the initial programme theory. This led to the identification of unintended consequences, which had not featured in the initial programme theory. The issue of unintended consequences was then pursued as the focus for the realist evaluation because these emerged as potentially important considerations for actors seeking to implement OPSS. Emergent CMO configurations around unintended consequences were first developed by GW, JS, JG, TS, LJ and FB and iterated through multiple co-investigator meetings, discussion with ASSIST’s advisory board of UK sexual health leaders and patient and public representatives and through feedback obtained from dissemination events.

### Patient and public involvement

The research team included a lay representative who contributed extensively throughout the project, shaping the design, methods and interpretation of data and is an author on this paper. Lay representatives were also included on the ASSIST Study Steering Committee and Expert Advisory Group, the Community Advisory Panel and the dissemination event.

## Results

There were three main perceived drivers to OPSS: reducing costs, maintaining sustainability of sexual health service delivery, and reducing inequities (Table 2, paper 3.1) [11]. We focused our realist programme theory on three, related unintended consequences arising from the introduction of OPSS into sexual health systems.

1. There may be difficulties in containing demand for OPSS
2. The introduction of OPSS may affect wider sexual health system sustainability, particularly when the context suddenly changes
3. The introduction of OPSS into sexual health systems may lead to widening of inequalities in sexual health.

Context-sensitive causal statements in the form of context-mechanism-outcome-configurations for each of these consequences are listed in Table 3 and visualised in supplementary file 1.

**Table 3:** Context mechanism outcome configurations, summarising how unintended consequences may arise through introducing OPSS and reinforcing or rebalancing reactions in the system

**1. Difficulties in containing demand** 1.1. When providers encourage users to use online services (C) and reduce the provision of clinic-based services (C) then there will be a demand for OPSS services (O) because service users have limited choice of services to choose from (M). 1.2. When COVID-19 severely limited availability of face-to-face clinic care (C) and made self-testing for COVID-19 widespread (C), service users became comfortable with trying OPSS (O) because remote healthcare & self-testing and sampling were normalised (M). 1.3. When an online service provision mimics online providers (e.g. online shopping) embedded in everyday life (C) service users become comfortable with trying OPSS (O) because of compatibility with their expectations and values (M). 1.4. When OPSS was able to meet the needs of service users (C), this caused high demand (O) because of convenience (M).
**2. Effects on wider sexual health system sustainability** 2.1. When clinics have to care for more complex cases but the reimbursement they get for such care is insufficient (C), clinics are at risk of closure (O) because they become a resource drain (M). 2.2. When funders with a reduced and fixed budget have to fund a new service such as OPSS (C) provision of other services (e.g. clinics) is likely to be reduced (O) because of affordability challenges (M). 2.3. When OPSS enables the management of ‘simpler’ cases outside of clinics (C) clinical staff face increasing job strain (O) because the complexity of cases presenting to clinics increases (M). 2.4. When OPSS enables the effective management of ‘simpler’ cases outside of clinics (C) junior staff in clinics face more administrative tasks and get reduced learning opportunities(O) and consequently potentially reduced job satisfaction (O) because of a reduced case-mix to learn from (M) and an unwanted role change (M). 2.5. When OPSS processes do not run as expected (C) service providers in clinics have additional work (O) because they are obliged to address service gaps (M). 2.6. When the OPSS processes do not run as expected (C) service users have additional work to contact services or attend clinics (O) because their needs are not addressed by OPSS (M). 2.7. When sexual health systems introduce or extend limits to OPSS access (C) determined users will find workarounds (O) because they highly value its relative advantage (M).
**3. Exacerbating inequalities in sexual health care** 3.1. OPSS requires people to have internet access and the ability to use the internet to order OPSS kits when face-to-face testing access is reduced (C). Individuals with a sexual health need that lack this access or capability are less likely to use OPSS and therefore less likely to test (O) because they do not have the means to order kits (M). 3.2. When people with a sexual health need do not have the opportunity to receive OPSS kits or do the tests in private (C) they are less likely to use or complete OPSS (O) and therefore less likely to test or receive results (O) because they perceive taking part may reveal information about their sexual health care need to others (M). 3.3. When service users with low resources are made to address their unmet sexual health needs in more challenging ways (e.g. blood needed to obtain STI/BBV results) amid reduced access to face-to-face services(C), they are less likely to complete all the steps required to receive a result or treatment if needed (O) because this places an unmanageable drain on their resources (M). 3.4. When some service users with unmet sexual health needs choose to use OPSS for testing (C), some of their other sexual health and wellbeing needs may not be addressed (O) because at present, online platforms cannot effectively replace a face-to-face conversation with a skilled sexual health advisor or clinician (M) 3.5. When information about the care provided to vulnerable people with STI/BBVs is not shared with other service providers (C) they may be less likely to receive all the care they need (O) because service providers are unclear about their care needs (M) and because those already vulnerable are least able to persist in seeking treatment not offered by services.
**4. Reactions in the system to reinforce and rebalance OPSS delivery** **4.1. With reference to CMO 1.4 reinforcing:** When commissioners observe high OPSS demand and satisfaction (O → C) they are more likely to divert more resources to it and expand its use (O) because they perceive it to be a success (M) **4.2. With reference to CMO 2.1–2.2 rebalancing**: When OPSS high demand results in higher costs for commissioners or providers than expected (C) they limit access to OPSS (O) because they seek to maintain the viability of clinic-based services (M) **4.3. With reference to CMO 3.1–3.4 rebalancing:** When providers or commissioners observe that some populations are less likely to take up or complete testing through OPSS (O → C) they encourage clinic attendance for these groups (O) because they think that face-to-face sexual health care may better address needs of some people at risk (M) and because face-to-face modalities fit with wider societal shifts back to face-to-face interactions (M).

### Difficulties in containing demand

**Fig. 1:**
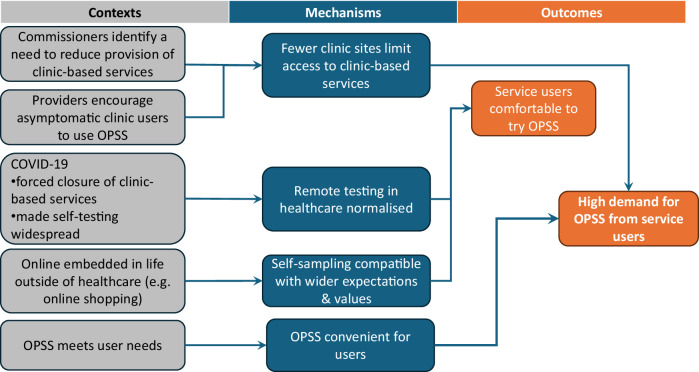
Summary of the Context-Mechanism-Outcome configurations and their linkages explaining why high demand may arise for OPSS

Establishment of OPSS followed tendering processes to re-commission both clinic-based and online sexual health services, which also led to a reduction in clinic-based services. In all cases, tendering negotiations started from a need to reduce costs due to reducing budgets, whilst meeting rising demand and unmet need in populations at higher risk of poor sexual health. It was assumed that introducing OPSS would enable cost-savings whilst freeing clinic capacity (Fig. 1). COVID-19 was a significant influence on responses to OPSS. Face-to-face clinic-based services were massively restricted during lockdown periods. Alongside this restriction in provision, remote healthcare, self-sampling, and self-testing became normalised due to COVID-19 restrictions and self-testing requirements. Both service users and staff reflected how OPSS became increasingly compatible with the shift of many aspects of life moving online outside of healthcare (Table 3, CMOs 1.1,1.2,1.3).

*“It became very popular very quickly and particularly during COVID and lockdown it became really embedded and patients really accepted this.” (CSA2, Service Manager, Workstream 3)*

Service users reported that when OPSS worked well, it was more convenient and accessible than clinic-based services [18], congruent with our initial programme theory (Table 3, CMO1.4). Indeed, surveillance data indicated an increase from 2.6% to 38.4% in the proportion of chlamydia testing that occurred online between 2015–2022 (Table 2, paper 1.1). The high demand was not just for previous users, but also for people that hadn’t used services before.

*“We rather hoped we could shift them onto the OPSS system and therefore allow those slots to go somewhere else in the clinic…. But a lot of the capacity, the online testing are new people who clearly would not be coming to the service otherwise.” (CSA1, Consultant, Workstream 3)* [11]

However, high demand was not always met. While in CSAs 2 and 3 testing volumes increased, in CSA1 testing did not increase to the same extent during and after COVID-19 lockdowns (Table 2, papers 1.1, 1.4). The difficulty in maintaining OPSS testing volumes during COVID-19 in CSA1 was in part because COVID-19 testing capacity was increased at the expense of OPSS testing. In CSA1, there were also delays in dispatching kits and providing service users with results (Table 2, paper 3.1). The result was also a decrease in user satisfaction [18]. Service users accustomed to online shopping (e.g. next day delivery) may have found the delays in OPSS particularly unacceptable (Table 3, CMO1.3).

*“I’ve ordered the kit online and I think it took three months for the kit to arrive and it’s been three or four weeks since I’ve done the test and I’ve still not had the results.”(Participant 78, White cis woman, aged 25–34*, Workstream 1*)* [19]

### Effects on sexual health service sustainability

While OPSS was intended to sustain sexual health service delivery, its introduction may have destabilised clinic services (supplementary file 1, Fig. 2). In CSA2, removing asymptomatic activity resulted in reduced income to clinic-based services but did not commensurately reduce workload (Table 3, CMO2.1).

*“The commissioners wanted to save money and in the sexual health tariff you get about from memory about £80, £90 per screen on top of whatever else you’re doing for the patient, […] unfortunately the knock-on effect of that was that patients, […] patients will do a screen through [OPSS] but they’ll still come to the service. But the way that the tariff was constructed didn’t account for [that], so it’s completely unsustainable from a financial point of view.” (CSA2, Consultant*, Workstream 3)

**Fig. 2:**
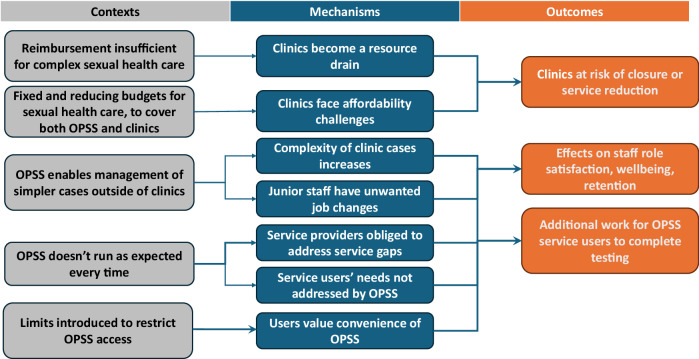
Summary of the Context-Mechanism-Outcome configurations and their linkages explaining why introduction of OPSS may affect wider sexual health system sustainability

The introduction of OPSS also affected staff workload in sexual health systems, and ensuing wellbeing. It increased the intensity of work for clinical staff who saw an increasingly complex caseload, placing strain on those staff and required a more skilled workforce.(Table 3, CMO2.3) Removing asymptomatic testing from clinic environments affected the workload of several staff groups, In particular, it limited opportunities for career development of healthcare support workers, a subset of junior staff who do not have professional clinical qualifications through reducing their contact with service users.(Table 3, CMO2.4) A change in workload was also experienced by some non-clinical staff whose roles changed to troubleshoot OPSS problems.(Table 3, CMO2.5)

*“My job has definitely changed quite a lot. So if we go sort of back to when the new contract came in, when the online testing started and to now, I don’t know how much of that is COVID, I don’t know how much the new contract is, and how much is online testing, but my role now seems to be seeing much more complicated patients.” (CSA3, Resident Doctor*, Workstream 3) [11]

*“[Healthcare support workers] lost their clinical role and I don’t think that is really acknowledged much. They are very unhappy, rightly so, because many of them were appointed on a clinical role and now many of them do an admin role.” (CSA2, Consultant*, Workstream 3) [11]

When OPSS did not work as intended, it could result in extra work for service users too, who then needed to contact services and potentially visit a clinic to get tested instead.(Table 3, CMO6) Clinic staff also reported that some service users went to significant effort to subvert rationing systems when OPSS was limited.(Table 3, CMO2.6)

*“Patients are trying to get round the issue that: oh well we can only have this amount of kits, we’ll just type our, spell our names slightly differently or put a different phone number on, and oh look we’ve got another record where we can have X amount of kits again.” (CSA3, Health Advisor*, Workstream 3)

### Exacerbating inequalities in sexual health

All CSAs sought to increase access and address recognised inequities in current provision when introducing OPSS. ASSIST quantitative analyses showed that the biggest increase in OPSS use was in MSM, who are at higher risk of STI/BBVs. However, proportional increases in testing (OPSS and clinic-based services) relative to pre-OPSS levels were greatest in sociodemographic groups at lower risk of STI/BBVs. People living in less deprived areas showed greater relative increases in testing activity, compared with pre-OPSS levels than those in the most deprived areas. Similarly, white British/Irish groups had larger relative increase in testing than other ethnicities [19]. (Table 2, papers 1.1, 1.3).

**Fig. 3:**
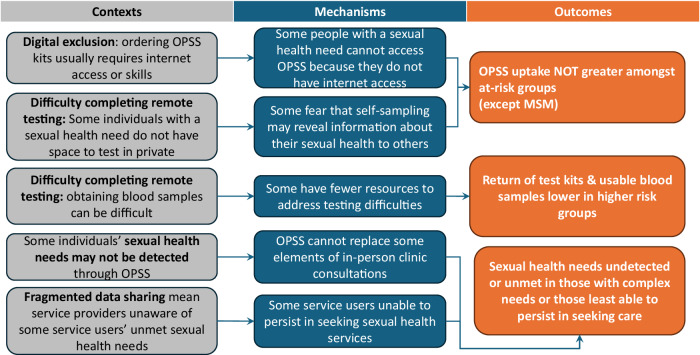
Summary of the Context-Mechanism-Outcome configurations and their linkages explaining why inequalities in sexual health may arise following the introduction of OPSS

We identified several mechanisms by which inequalities may be widened by introducing OPSS into the sexual health economy (Fig. 3). People who are digitally excluded or with low health literacy could not order OPSS kits online. Moreover, alongside introduction of OPSS, many clinics reduced services and/or offered online appointments or triaged patients online, particularly when walk-in services were restricted. Therefore, staff were concerned that, with the first point of access often being online across the whole sexual health economy, individuals that were digitally excluded and/or with low health literacy would be less likely to be tested for STI/BBVs and their sexual health needs unmet.

*“Those people who have the means and the technology to get on the Internet, it’s really beneficial, but there’s always a question about those people who don’t have the means to get onto the Internet.” (CSA1, Nurse*, Workstream 3)

For those that did access OPSS, inequalities may still arise when testing is not completed. Howarth et al. [19] described socio-demographic variations in return of processable samples from OPSS consistent with widening inequalities, e.g. individuals living in least deprived areas were more likely than those in most deprived areas to return processable samples [19]. Some service users were more able than others to complete self-sampling and some required help [18].

*“It was very hard to get the blood out of. So I just ended up binning it because it was a nightmare.”(Participant 19, White cis man, aged 35–44*, Workstream 1) [18, 19]

We theorise that structural factors placing some people at greater risk of unmet sexual health needs may also hinder their capacity to complete testing, particularly when they experience difficulties. These individuals may have limited private space for self-sampling (Table 3, CMO3.2). They are less likely to have the flexibility to take time off work to attend clinics if they are not able to take a blood sample themselves. They may also have lower health literacy or fewer networks that can help resolve difficulties in obtaining samples (Table 3, CMO3.3).

Inequalities may arise if risks to service users’ sexual and reproductive wellbeing are not identified or addressed through an OPSS interaction. Staff were concerned that while service users may present with a simple need for STI testing, in a face-to-face interaction with a skilled sexual health advisor or clinician, other needs can be identified and addressed opportunistically (e.g. vaccination, prevention, contraception) (Table 3, CMO3.4).

*“We audited our asymptomatic screening clinic a few times and 30% needed to be seen by a nurse or a doctor. So that may be that they had a safeguarding vulnerability that needed to be actioned or some symptoms.” (CSA2, Manager*, Workstream 3*)*

Finally, a lack of interoperability between some OPSS data systems and clinic-based providers could mean that some individuals testing positive for STI/BBVs through OPSS are not offered treatment and partner notification (contact tracing), and this omission goes undetected. Aligned to candidacy theory, service users that are already vulnerable e.g. because of adverse living conditions, poverty, lack of familiarity with England’s national health system, may be least able to persist in seeking treatment if services do not offer it (Table 3, CMO3.5).

### Reactions in the system to reinforce or rebalance OPSS delivery

COVID-19 led to different effects on OPSS delivery in CSAs (Table 2, CMO 4.1-4.3). In CSA2, STI testing was maintained and increased through OPSS. Since 2020, the scope of OPSS was widened to include users with mild symptoms and those whose partners had tested positive for an STI/BBV. Clinic staff also referred more clinic service users to OPSS during this time. Its success during COVID-19 was used as evidence to extend the OPSS contract (Table 3, CMO4.1).

*“COVID-19 restrictions resulted in a large increase in activity in the service as local … clinics encouraged many patients who would usually attend clinics to test online…The resulting underspend in clinics will be used to fund the increased costs of the online service.” (Document 109, 2021, CSA2. Paper seeking extension to renew OPSS contract*, Workstream 3*)*

In contrast, in CSA3, the sudden shift of almost all activity to OPSS in 2020 resulted in much higher OPSS costs than anticipated, and savings had to be found from clinic services. The resulting risk to clinic sustainability led to prolonged negotiations with commissioners to restrict eligibility (Table 3, CMO4.2). It took two years to implement this restriction.

*“I think we’d been asked to move maybe 30% of our screening online whereas we went to probably like 95% of our screening being online overnight because of Covid and then to take that away from people is then really difficult because it’s already out there.” (CSA3, Service Manager*, Workstream 3) [11]

There was a slight drop in OPSS activity nationally in 2022 relative to clinic activity compared with 2020 and 2021 [20]. Some clinic staff described activities to promote face-to-face testing again. These were in part responding to the mpox epidemic, where in-person attendances for vaccinations led to increased face-to-face sexual health service delivery opportunities for vulnerable service users. They were also to attract groups they suspected were better suited to in-person care, also reflecting wider societal shifts recognising benefits of face-to-face interactions since COVID-19.

*“Looking at the more vulnerable groups … on outreach so homeless, black, and ethnic minority groups and commercial sex workers so looking at other vulnerable groups…. what we’re trying to do at the moment is create more face-to-face appointments.” (CSA2, Manager*, Workstream 3*)*

## Discussion

This synthesis across multiple sources of evidence found unintended consequences from the introduction of OPSS for STI/BBV. Whilst OPSS was cheaper per test than clinic testing, high and uncontrolled demand could have resulted in higher total costs than planned and could jeopardise the sustainability of wider sexual health systems. While OPSS was highly acceptable to many service users, it may have increased inequalities in sexual health. Changes, in reaction to these consequences, led to both attempts to both achieve OPSS expansion in one area and rebalancing of clinic-based and OPSS activity in others.

### Strengths and limitations

The strengths of this study included the application of realist methodology which is well suited to a rapidly changing context [10]. It combined multiple empirical qualitative and quantitative data sources including extensive lived experiences of OPSS from diverse perspectives – sexual health service users, providers, funders and researchers - from across the UK.

Limitations to the evidence informing this synthesis include the lack of inter-operability between clinic-based and OPSS data systems, meaning it was not possible to track the outcomes for all OPSS service users. It was also not possible to measure set up costs. These limitations inevitably affect the robustness of our conclusions.

### Comparisons with other literature

We describe consequences arising from the introduction of OPSS as “unintended.” However, some consequences were not unforeseen. For example, Topol’s [21] report on online delivery in health care predicted staff roles would change and identified a need to adapt to more clinic complexity. This increase in clinic complexity also reported by Turner et al. [4] in their evaluation of the introduction of OPSS within an English region [4]. The review warned of increasing health inequalities [21]. Our findings, suggesting widening of some sexual health inequalities, are also congruent with Sumray et al. [3] and Iyamu et al. [22] which found higher OPSS uptake among white people in higher socioeconomic circumstances [3, 22]. Our study proposes several mechanisms by which health inequalities can arise following the introduction of digital sexual health interventions, beyond differential uptake of testing due to differences in access to technology or digital literacy.

There is widespread recognition that OPSS should be a complementary option integrated within existing sexual health services [6, 23]. This synthesis theorises how unintended consequences can develop when OPSS is not fully aligned with clinic-based sexual health care. However, ensuring OPSS is fully integrated with clinic-based services (i.e. clear pathways between them, shared data, capacity across both balanced) may not be sufficient if it comes into conflict with other healthcare priorities (i.e. competing demands for testing facilities during the COVID-19 pandemic).

Our findings have arisen at a time when the role of asymptomatic testing for chlamydia and gonorrhoea across all populations is being increasingly questioned, given the lack of evidence that it contributes to reducing prevalence or long-term sequelae, and concerns about the increasing risk of antimicrobial resistance [24, 25]. As studies within ASSIST have demonstrated, OPSS is a vehicle for increasingly the volume of screening delivery [12], but this study highlights its unintended consequences, further adding to the debate about the public health benefits if asymptomatic testing for chlamydia and gonorrhoea.

In evaluating OPSS models, outcomes of quality (processible results, time to diagnosis and treatment) and equity (by individual characteristics) should be included, not just testing volumes. Standards should consider OPSS and other online services as part of integrated sexual health systems rather than as separate entities [26].

## Conclusions

Unintended consequences following the introduction of OPSS into sexual health systems included high and uncontrolled demand which could result in higher total costs than planned and could destabilise wider sexual health systems. While OPSS was highly acceptable to most service users, clinic-based services may better meet the needs of some people. Rebalancing clinic-based and OPSS activity is needed to address the effects of OPSS on the wider sexual health system and changes in context.

These findings have relevance not only to sexual health systems implementing OPSS, they have wider applicability for health service delivery, given plans in many countries including England to digitalise large sectors of healthcare [27]. Firstly, we recommend that those planning and implementing OPSS (or other digital transformations) consider the whole healthcare system. Complementarity between face-to-face and digital technologies is most achievable when a systems perspective adopted from the outset, and when there are processes to calibrate and recalibrate levels of delivery across face-to-face and online platforms. Secondly, accurate data are needed across the whole service user pathway for comprehensive evaluation of the costs, impacts and inequalities following OPSS. These data are needed to make informed decisions about reaching the most equitable balance of face-to-face and online delivery. Thirdly, assessing the effectiveness of OPSS on population health needs to include standardised quality and equity measures, not just volumes of care delivery.

## Supplementary information

Additional File 1 **Slide 1**. Initial ASSIST logic model, developed 2020 (adapted from Gibbs et al. 2022). **Slide 2**. Initial programme theories to surface assumptions about OPSS and how it might achieve its outcomes. **Slide 3**. Identification of unintended consequences of OPSS related to ASSIST initial logic model. **Slides** **4-6**. Early context-mechanism-outcome-configurations (CMOCs) considering OPSS as a digital innovation

## Data Availability

This paper is based on data analysed as part of ASSIST. Findings are available in the following publications: -Spence T, Gibbs J, Wong G, Howarth A, Copas A, Crundwell D, et al. Evaluating the Implementation of Online Postal Self-Sampling for Sexually Transmitted Infections in England: Multisite Qualitative Study. J Med Internet Res. 2025;27:e72812.-Gibbs J, Stirrup O, Tostevin A, Howarth A, Dewsnap C, Ross JDC, et al. Sexually transmitted infection testing and key outcomes following implementation of online postal self-sampling into sexual health services in England: a retrospective observational study of routinely collected service-level healthcare data. Lancet Reg Health Eur 2026;61:101541.-Spence T, Howarth A, Reid D, Sheringham J, Apea V, Crundwell D, et al. How does online postal self-sampling (OPSS) shape access to testing for sexually transmitted infections (STIs)? A qualitative study of service users. BMC Public Health. 2024;24:2339.-Howarth A, Harb A, Mohammed H, Burns F, Estcourt C, Bloch SCM, et al. Uptake, positivity, and equity of online postal self-sampling for chlamydia testing in England: a retrospective cohort study. Lancet Reg Health Eur. 2025;15;56:101412.-Gibbs J, Howarth A, Tostevin A, Tittle V, Stirrup O, Mercer C H, et al. Factors associated with the online postal self-sampling testing cascade in England, 2022. Sex Transm Infect. 2026. In press. The raw data that further support the findings of this study are not publicly available, as participants did not consent to this. Questions about the data can be directed to the corresponding author Jessica Sheringham ([j.sheringham@ucl.ac.uk])

## List of abbreviations

ASSIST: Assessing the impact of online postal self-sampling for sexually transmitted infections on health inequalities, access to care and clinical outcomes in the UK
BBV(s): Blood borne virus(es)
CMO: Context Mechanism Outcome
COVID-19: Coronavirus disease
CSA: Case study area
Mpox: Official name for the virus formerly called monkeypox
MSM: Men who have sex with men
NHS: National health service
NIHR: National Institute for Health and Care Research
NPT: Normalisation process theory
OPSS: Online postal self-sampling
PIs: Principal investigators
STI(s): Sexually transmitted infection(s)
