# Supplementary material for: The impacts of introducing online postal self-sampling for sexually transmitted infections on the sustainability and equity of sexual health systems: lessons learned from a multi-method UK-wide realist evaluation
Source: BMC Med. 2026 May 18;24:388. doi: 10.1186/s12916-026-04908-7 (PMC13352629; doi:10.1186/s12916-026-04908-7)
Supplement: Supplementary file 1 — Additional File 1 Slide 1. Initial ASSIST logic model, developed 2020 (adapted from Gibbs et al. 2022). Slide 2. Initial programme theories to surface assumptions about OPSS and how it might achieve its outcomes. Slide 3. Identification of unintended consequences of OPSS related to ASSIST initial logic model. Slides 4-6. Early context-mechanism-outcome-configurations (CMOCs) considering OPSS as a digital innovation [file 12916_2026_4908_MOESM1_ESM.pdf]

Initial ASSIST logic model, developed 2020 (adapted from Gibbs et al, 2022)

CONTEXT & SYSTEM HISTORIES		Outcomes: short	medium	impact
		Decrease health inequalities		
Health and Social Care Act – change in sexual health commissioners to local authorities	Developments in tech make OPSS at scale feasible	↑ HIV testing<br>↑ STI testing<br>↓ time to results	↓ untreated	↑ patient experience
Policy: promotion of digital self management strategies	Drive to ‘channel shift’ asymptomatic F2F users → OPSS	Shift of asymptomatic users to OPSS	↓ demand for F2F	↓ transmission
Behaviour/epidemiology: ↑demand for sexual health services	Public expectation for online access, self-sampling		Change in case mix	↑ s health and wellbeing
Economics: Disinvestments in sexual health services		New clinical pathways	↑ provider and patient satisfaction	↑ efficiency and quality of services
			Inform guidance and standards	↑ cost effectiveness

Initial programme theories, formed in ASSIST co-investigator workshop (October 2021)

Service users

- Easy to find and access
- Convenient
- Easy to use (fits in with 21st Century life)
- Provides privacy and minimises embarrassment / judgment by others
- Easy to do, and people are willing to do it
- People believe the results of the self-sample
- As good as face to face

Commissioners and providers

- Saves money
- Everyone has equal access
- Frees up capacity (efficient way to manage asymptomatic service users)
- Saves time for providers
- Provides the additional capacity to deal with demand

ASSIST logic model – flagged where unintended consequences arose

CONTEXTS	Enablers	Outcomes: short	medium	impact
			**Decrease health inequalities** (2)	
Health and Social Care Act – change in sexual health commissioners to local authorities	Developments in tech make OPSS at scale feasible	↑ HIV testing<br>↑ STI testing<br>↓ time to results	↓ untreated	↑ patient experience
Policy: promotion of digital self management strategies	Drive to 'channel shift' asymptomatic F2F users → OPSS	Shift of asymptomatic users to OPSS	↓ demand for F2F	↓ transmission
Behaviour/epidemiology: **↑demand for sexual health services** (1)	Public expectation for online access, self-sampling		Change in case mix	↑ s health and wellbeing
Economics: Disinvestments in sexual health services		New clinical pathways	**↑ provider and patient satisfaction**	**↑ efficiency and quality of services** (3)
			Inform guidance and standards	↑ cost effectiveness

1. Early CMOCs on demand (discussed with co-investigators and advisory board, May and July 2024, wider stakeholders in ASSIST symposium September 2024)

(Initial PT: "OPSS is Easy to find and access" & "saves money") → **<u>unmet</u> demand for F2F + normalisation of home testing → difficulties in controlling demand for OPSS**

Context	Mechanisms	Outcome	Unforeseen consequences
**F2F not convenient**: takes a long time /requires travel, disruption to routine	OPSS increases **convenience** of getting tested	High demand amongst STI service users for OPSS ✓	In CSA1, increase in demand alongside capacity problems during COVID **reduced timely/safe delivery of OPSS** (**x increased** time to results)
**F2F not accessible**: Getting clinic appointment for STI testing is difficult (before and after COVID-19 lockdowns)<br>Services were **not available** in clinics during COVID-19 lockdowns	Getting tested **available** only through OPSS	OPSS testing uptake is high	*SH wellbeing **not** increased?*<br>Risk of microbial resistance, harm from diagnosis?
Seeking testing for an STI through clinics can feel **stigmatising**	OPSS increases possibility of **privacy** in STI testing		OPSS **costs more than expected (in CSA3)** (**x** provider satisfaction)
COVID-19 **normalises at-home testing**		↓ demand for F2F ✓	OPSS **undermines financial model of SHS** (**x** service efficiency)

✓ = congruent with initial logic model or programme theory assumptions

x = congruent with initial logic model or programme theory assumptions

2. Early CMOCs on SHS efficiency (discussed with co-investigators and advisory board, May and July 2024, wider stakeholders in ASSIST symposium September 2024)

(initial PT: "frees up capacity") → OPSS affects the "work" of **all** actors in 'the system' – sometimes destabilising the wider sexual health system

Context	Potential mechanism	Outcome	Unforeseen consequences
- For commissioners since HASCA, large number of commissioner – provider contracts ✓	Changed the commissioning pathway. Fewer commissioner – provider contracts		- More provider – provider subcontracts (in 2 CSAs)<br>- **Diverted clinic staff** to managing OPSS delivery/troubleshooting (in 1 CSA) (x)
- For providers – work due to low-risk testing meant full clinics ✓	OPSS changes the testing pathway ✓	Change in clinic case mix ✓<br>Difficulties in blood self sampling<br>Delays in getting results (x)	- Higher complexity in clinics makes **work harder for clinicians**<br>- ↓ **opportunities for training and career development** of junior staff (x)
- For service users (link to convenience in theme 1). Clinic-based testing was time consuming and difficult	Delays or limits in testing capacity introduce new work for service users		Work for service users to<br>- **Visit clinics** when they can't give blood<br>- **'game' the system** to 'beat' capping (ie testing limits), e.g. invent new user profiles, schedule orders for midnight on new day<br>- **check up on test results**/re-ordering kits

✓ = congruent with initial logic model or programme theory assumptions

x = congruent with initial logic model or programme theory assumptions

3. Early CMOCs on inequalities post OPSS (discussed with co-investigators and advisory board, May and July 2024, wider stakeholders in ASSIST symposium September 2024)

(initial PT: "everyone has equal access") → differences in acceptability, and use of OPSS lead to a risk of increasing inequalities in access to appropriate sexual health care

Context	Potential mechanism	Outcome	Unforeseen consequences
Commissioners in all sites sought OPSS to reduce inequalities ✓	Some users found OPSS more **acceptable/easier** than others	Some groups **didn't use OPSS** as much as others<br>Some **high users of OPSS not typically high risk** (e.g. white females)	Inequalities may widen **x**
Some population groups are more at risk of and from STIs than others	Some users find **elements of OPSS** (e.g. blood testing) hard	OPSS **treatment cascade** suggests: Ethnic minority groups and those living in disadvantaged areas are LESS **likely to receive treatment** than others	
	OPSS algorithms can't pick up risks that can be explored in F2F clinic appointments	Some individuals using OPSS have **risks that remain unmanaged**	

✓ = congruent with initial logic model or programme theory assumptions

x = congruent with initial logic model or programme theory assumptions
